# Intraluminal visualization of appendiceal hemorrhage with the digital single-operator pancreatobiliary scope: a case report

**DOI:** 10.1055/a-2860-7398

**Published:** 2026-06-01

**Authors:** Lina Xiao, Yan Ou, Chuanhua Yang, Jingxian Yu, Yun Chen, Chunhui Wang, Juan Liao

**Affiliations:** 1 Department of GastroenterologyWest China Hospital of Sichuan UniversityChengduChina; 2 Department of Gastroenterology 618726 Sichuan University West China Fourth HospitalChengduChina; 3 Department of General Surgery 618726 Sichuan University West China Fourth HospitalChengduChina; 4 Department of Pathology 618726 Sichuan University West China Fourth HospitalChengduChina

**Fig. 1:**
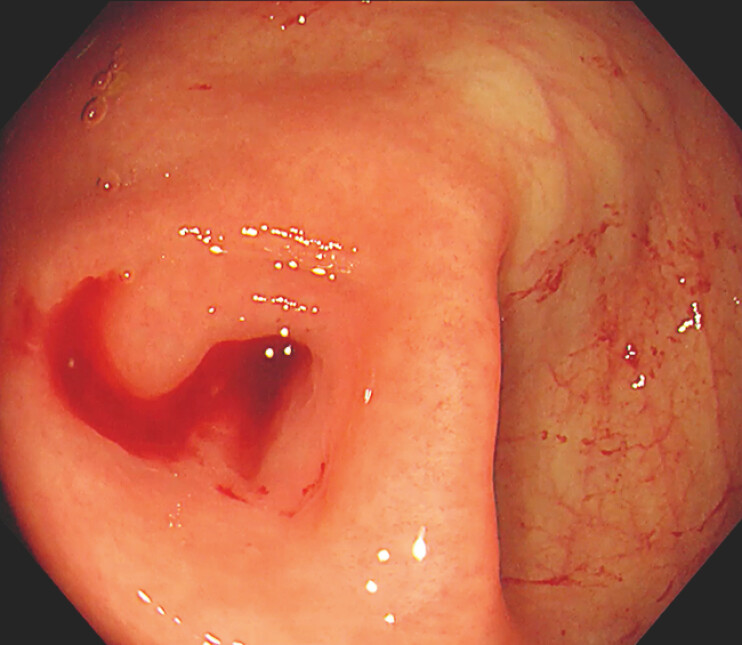
Fresh blood oozing from the appendiceal orifice.

**Fig. 2:**
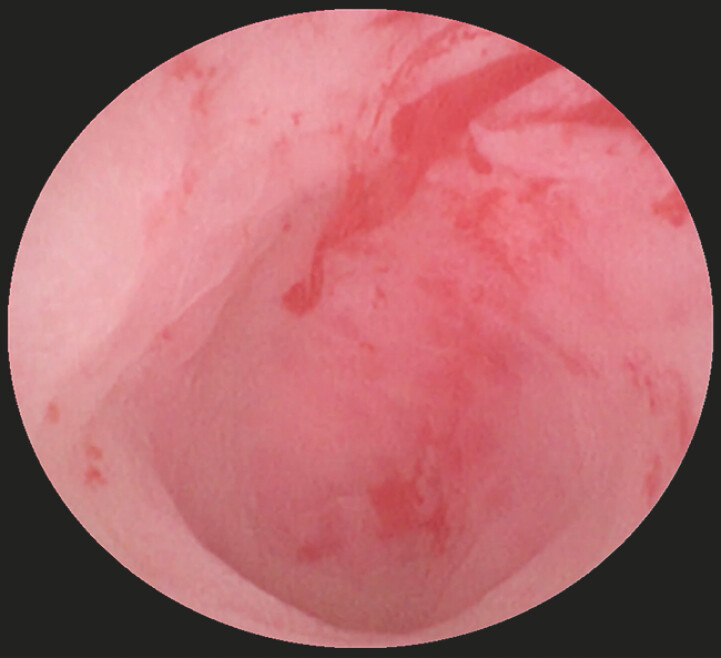
Focal mucosal erosion in the appendiceal lumen with active oozing.

**Fig. 3:**
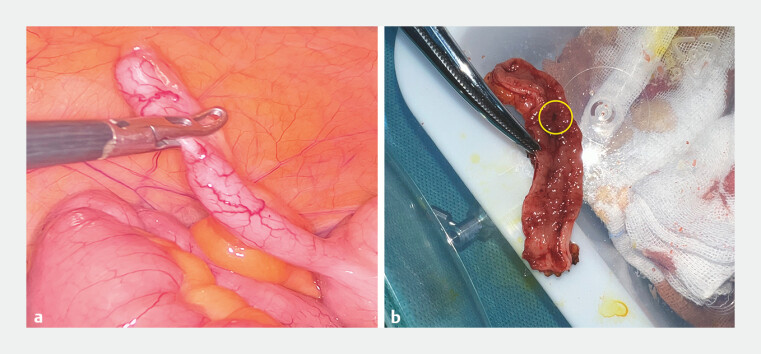
The surgical specimen from the patient with appendiceal hemorrhage. **a** A laparoscopic view showing the normal shape and color of the appendix. **b** Gross dissection of the appendiceal specimen revealing clot adhesion on the focal mucosal erosion.

**Fig. 4:**
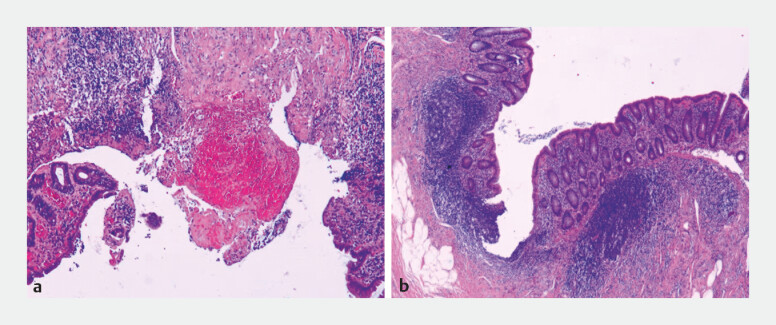
Postoperative histopathology of the resected appendiceal specimen. **a** Focal mucosal erosion with associated clot formation. **b** Intact and well-demarcated mucosal layers of the appendix at non-bleeding sites.

A 34-year-old man was admitted with a 10-hour history of intermittent hematochezia. On admission, his vital signs were stable; physical examination showed no abdominal tenderness, distension, or abnormal bowel sounds. Routine hematological tests showed a normal hemoglobin level (15.3 g/dL). Emergency colonoscopy revealed bleeding from the appendiceal orifice ( Fig. 1 ). After endoscopic irrigation to clear residual blood, bleeding persisted as oozing ( Video 1 ). Then, we used a digital single-operator pancreatobiliary scope (9 Fr) to visualize the appendiceal lumen, revealing mucosal erosion with active oozing of fresh blood ( Fig. 2 ). Laparoscopic appendectomy was subsequently performed for hemostasis ( Fig. 3 ), and hematochezia completely resolved postoperatively. Pathological examination of the resected specimen confirmed focal mucosal erosion and clot in the appendix ( Fig. 4 ), consistent with the endoscopic diagnosis.

Colonoscopy revealed bleeding from the appendiceal orifice, and visualization of the appendiceal lumen using the digital single-operator pancreatobiliary scope demonstrated focal mucosal erosion with active oozing.Video 1

Appendiceal hemorrhage is a rare etiology of lower gastrointestinal bleeding. Although digital subtraction angiography and computed tomography angiography aid in the diagnosis of appendiceal hemorrhage 1 2 , these modalities only work in patients with overt active bleeding 3 . The use of an ultrathin gastroscope for intraluminal imaging of appendiceal bleeding has been reported previously 4 . However, compared with the digital single-operator pancreatobiliary scope – featuring a smaller caliber (≤3.2 mm caliber) – ultrathin gastroscopes require more proficient endoscopic expertise and a relatively dilated appendiceal lumen for successful intubation.

To our knowledge, this is the first case using a digital single-operator pancreatobiliary scope for intraluminal visualization of appendiceal hemorrhage, enabling easy access to narrow lumens and precise identification of bleeding sites/causes to guide diagnosis and treatment.

Endoscopy_UCTN_Code_CCL_1AD_2AF
